# Bacteria and Septicæmia

**Published:** 1876-02

**Authors:** 


Bacteria and Septicæmia.—In an elaborate experimental paper (*New York Medical Record*), Dr. T. E. Satterthwaite arrives at the following conclusions:

1. Bacteria are certain vegetable organisms which belong probably to the algæ; they are found abundantly in nature, but chiefly where there is moisture.

2. They exist in the body in health, covering the mucous membranes from the mouth to the anus, and sometimes appear to penetrate a certain distance into the system, without causing symptoms of disease.

3. They also exist in putrefying fluids, and in various disease-processes, occurring in hot and cold abscesses, in the blebs of erysipelas, and in simple blisters.

4. It is doubtful whether the virulent principle of infective diseases is albuminous.

5. This principle does not reside in the perfectly clear fluid that passes through porous clay. In putrid infectious fluids this appears to be certain. The poison is rendered less virulent by repeated filtrations through common filter paper.

6. The virulent principle may be boiled for hours, filtered numbers of times in the ordinary way, boiled with alcohol, and again filtered and dried, and yet the watery extract of such a dry residue will produce septic symptoms. It is therefore soluble, or at least suspended, in water.

7. The liquid which is thus poisonous may be clear to the eye, but contains granules under the microscope.

8. These granules have not produced bacteria in a number of instances when they were placed in a suitable condition to do so.

9. We cannot, therefore, feel that satisfactory evidence has been brought to show that, in any of the diseases or processes enumerated, minute organisms are the sole and sufficient causes of disease.

